# Near-infrared light-induced transcranial photobiomodulation enhances the visual pathway’s function in healthy volunteers

**DOI:** 10.1117/1.JBO.30.S2.S23908

**Published:** 2025-08-26

**Authors:** Alex O. Trofimov, Elizabeth Kalinkina, Anastasia Medvedeva, Ekaterina Volkova, Anastasia Kivenko, Edwin M. Nemoto, Olga A. Bragina, Denis E. Bragin

**Affiliations:** a Privolzhsky Research Medical University, Department of Neurological Diseases, Nizhny Novgorod, Russia; b University of New Mexico, Department of Neurology, School of Medicine, Albuquerque, New Mexico, United States; c Lovelace Biomedical Research Institute, Albuquerque, New Mexico, United States; d New York Medical College, Department of Physiology, Valhalla, New York, United States

**Keywords:** near-infrared photobiomodulation, eye tracking, visual pathway’s function

## Abstract

**Significance:**

Low-level near-infrared light-induced transcranial photobiomodulation (NIR-TPBM) is a promising technology for improving cerebral blood flow and metabolism. However, the effects of NIR-TPBM on the visual pathway’s function remain poorly understood.

**Aim:**

The aim was to assess the visual pathway’s function changes in response to NIR-TPBM in young, healthy volunteers.

**Approach:**

This single-center, randomized, controlled clinical trial included 98 healthy volunteers with a median age of 23.2 years. Participants were randomly assigned to two groups: NIR-TPBM (18 men and 30 women) and sham NIR-TPBM (19 men and 31 women). Eye-tracking procedures were conducted in both groups before and after either PBM or sham intervention. In the NIR-TPBM group, low-fluence NIR-TPBM was applied to the left and right fronto-temporal regions using a NIR-TPBM device (Elmedlife H^®^, Nizhny Novgorod, Russian Federation). In the sham group, participants wore the NIR-TPBM helmet, but the NIR-TPBM mode remained deactivated. The duration of the sessions was identical for both groups. Changes in visual pathway function were evaluated by analyzing the dynamics of vertical and horizontal ocular vergence reactivity indices (VRx) using the EyeTracker application (BVG Software Group LLC, San Francisco, California, United States) on iPadOS 16 before and after the intervention. Statistical analysis was performed using nonparametric methods, with a significance level set at p<0.05.

**Results:**

NIR-TPBM procedures led to a significant increase in both vertical and horizontal VRx values. Post-procedure vertical VRx was significantly higher than pre-procedure values (0.879 [0.761; 0.918] versus 0.774 [0.721; 0.929], p<0.001). Similarly, horizontal VRx increased significantly after NIR-TPBM compared with baseline (0.943 [0.848; 0.969] versus 0.772 [0.651; 0.890], p<0.001). No adverse effects were observed during or after the NIR-TPBM sessions.

**Conclusions:**

NIR-TPBM enhances visual pathways function by increasing both vergence reactivity indices in young healthy volunteers.

## 1 Introduction

**Fig. 1:**
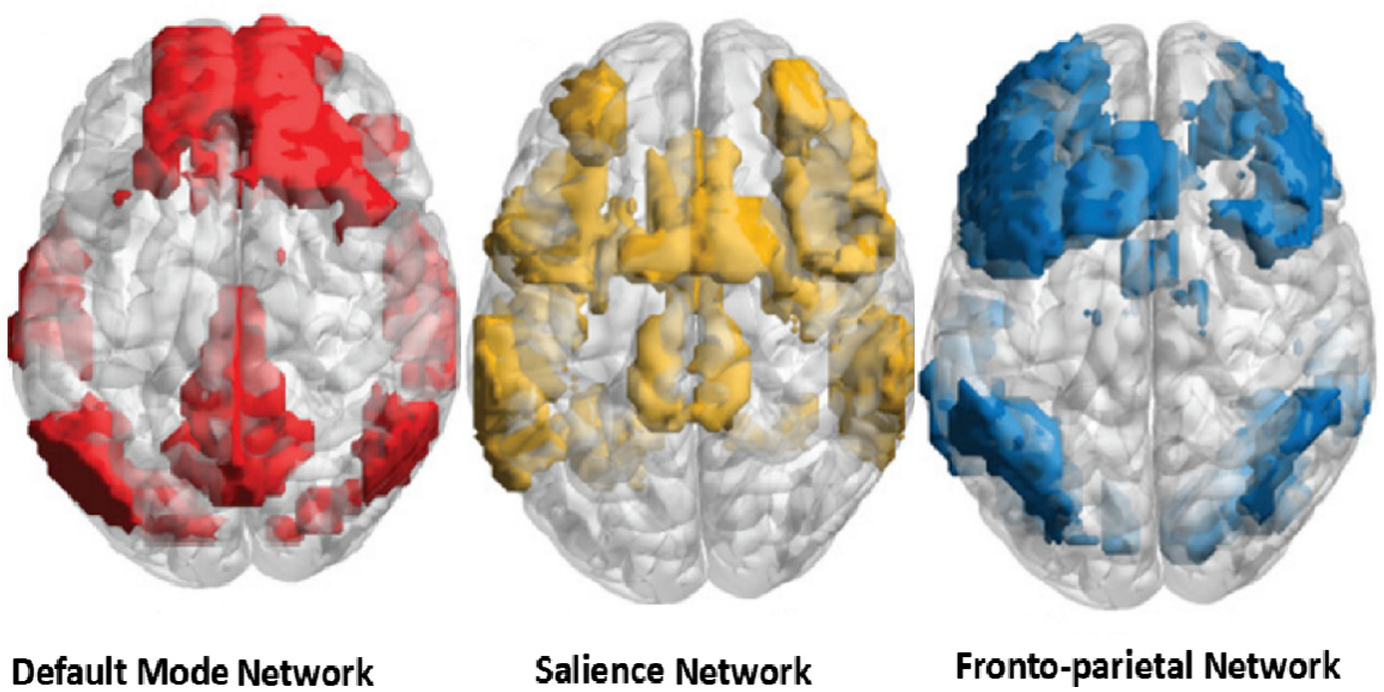
Proposed locations of the default mode network (red regions), salience network (yellow regions), and frontoparietal network (blue regions). Modified from Schimmelpfennig et al.^14^

Low-level near-infrared light photobiomodulation (PBM) was first proposed for local wound treatment in the mid-1960s by Endre Mester and other National Aeronautics and Space Administration researchers.^1^ Subsequently, its effectiveness was demonstrated for a variety of pathologies, both clinically and experimentally, in the red (600 to 670 nm) and near-infrared (NIR) (800 to 1100 nm) spectrum. As was shown later, the effects of PBM are mediated by the absorption of photons by mitochondrial cytochrome-C-oxidase (CCO), leading to an increase in the synthesis of adenosine triphosphate molecules. In addition, the effect of NIR light on the vascular endothelium causes photodissociation of nitrous oxide and leads to regional vasodilation,^2^ which explains the beneficial effects of PBM in a wide range of extra- and intracranial diseases and injuries.^3^^–^^7^ Although the effect of PBM on deoxyribonucleic acid and the genomic target pool in stem cells is considered as one of the mechanisms of long-term effects,^8^ this is not recognized by all researchers.^9^ The effect of photon energy is believed to be limited to the CCO of neurons, which causes immediate changes in their activity.^10^ However, according to Hennessy and Hamblin,^11^ the depth of cerebral penetration of NIR light can reach 4 to 5 cm, which allows modulation of not only the function of cortical structures but also of subcortical and conducting pathways of the brain. Moreover, the modulating effects of NIR light in healthy people are difficult to explain solely by an increase in oxygen consumption and regional metabolism because this occurs only during stimulation.^3^ At the same time, it is known that pools of neurons in the brain are interconnected into larger structures called neural networks. To date, many such networks have been described,^12^^,^^13^ and although the functions of some of them remain underinvestigated, the functioning of others is well understood. The latter comprises five networks called “canonical,” three of which are directly involved in the sensorimotor regulation of oculomotor activity and visual perception. These are the default mode network (DMN), the frontoparietal network (FPN), and the salience network (SN), and the putative locations of which are shown in Fig. 1.

The functional modulation of these networks causes remodeling of sensory perception and motor response, which may underlie the short-term effects of brain stimulation.

This was confirmed by Wang et al., who revealed changes in the spectral activity of the brain in the alpha and beta frequency ranges during near-infrared light-induced transcranial photobiomodulation (NIR-TPBM) in healthy subjects. EEG changes were observed not only in the NIR-TPBM zone, coinciding with the localization of FPN, but also in the domain of the contralateral parietal–occipital network involved in visual perception processes.^15^ Experimental studies on mice have also shown immediate positive effects of NIR-TPBM on spatial perception processes.^16^^,^^17^ Similar effects have been described in clinical studies.^18^

Thus, existing data suggest that NIR-TPBM has a much more profound effect on brain function and can likely modulate visual perception and eye movement coherence.

Significant progress in these studies has been achieved using pupillometry, a non-invasive method for assessing pupil dilation, which, however, does not allow one to judge the synergy of eye movements during longitudinal object tracking.^19^ At the same time, the use of eye tracking has an undeniable advantage over pupillometry because it allows for evaluating not only the dynamics of the pupils but also the compatibility of oculomotor activity, especially when assessing the effectiveness of transcranial stimulation (modulation) of the brain.^10^ Particularly, the changes in oculomotor conjugation and visual pathway function based on eye-tracking parameter dynamics in response to NIR-TPBM remain poorly understood and require study.

The aim of this work was to evaluate changes in the visual pathway’s function in response to NIR-TPBM in young, healthy volunteers.

## 2 Material and Methods

### 2.1 Design and Study Population

This single-center, randomized, controlled clinical trial adhered to the standards of the Declaration of Helsinki. The design was approved by the Local Ethics Committee and included ninety-eight right-handed white healthy volunteers from September 2023 to July 2024 (37 men and 61 women; median age was 23.2 years, min 22, and max 24). Subjects were randomly assigned using the Research Randomizer program^20^ into two groups: first group—“true” NIR-TPBM (18 men and 30 women); second group—sham (19 men and 31 women). Envelopes generated by a registrar not involved in registration and examination were activated immediately after registration of a volunteer who did not know whether they had received NIR-TPBM. The groups were matched in gender and age.

Informed consents were obtained from all subjects. All study participants were free of any neurological (cognitive impairment, bipolar disorder, depression, cerebrovascular disease or traumatic brain injury history, alcohol or drug addiction disorder, etc.) or ophthalmological disease (including myopia, hypermetropia, astigmatism, and strabismus). All subjects had a visual acuity of 1.0.

### 2.2 Eye Tracking Protocol

**Fig. 2:**
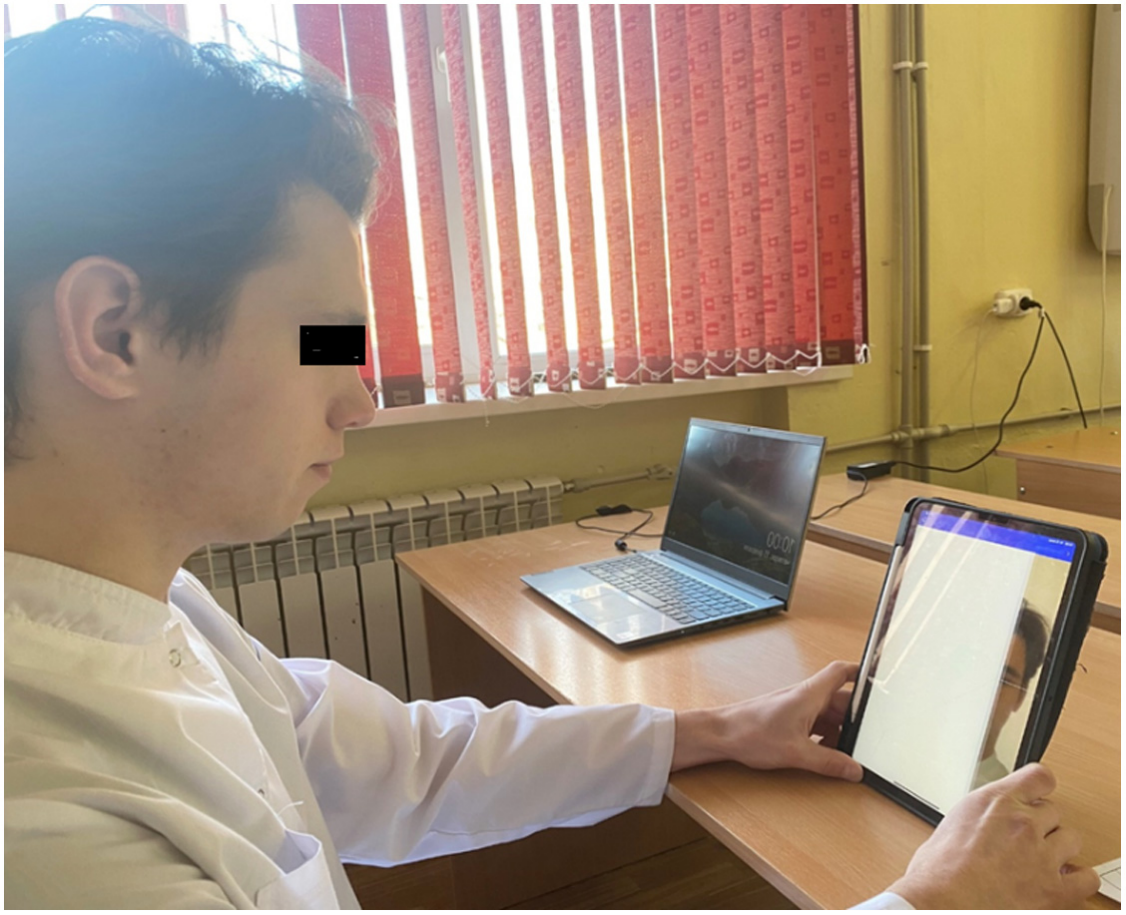
Overview of the eye-tracking procedure.

The eye tracking procedure was done as described earlier^15^ in both groups before and after NIR-TPBM or sham procedures. The eye tracking procedure was conducted under the supervision of a senior investigator (AT), who positioned the subject’s head to determine pupils throughout the study. A round marker with a diameter of 3 mm, changing its color from white to red with a frequency of 1 Hz, moved randomly across the 13’ screen of iPad 16 Pro (Apple, Cupertino, California, United States). The subject was asked to follow it with his or her gaze. At the same time, the position of the center of the pupils was estimated using the iPad 16 application Eye-Tracker (BVG Software Group LLC, San Francisco, California, United States) (Fig. 2).

The sampling frequency of the assessment was 20 Hz. Frames with incorrectly recognized pupils, closed eyes due to blinking, or other artifacts were automatically detected and removed. The analysis focused on the best 300 consecutive eye-tracking frames, with the entire procedure lasting 45 s. At each time point, the tracker computed the angular velocities of both eyes separately in the vertical and horizontal directions (measured in radians per second). These values included left vertical velocity (LVV), right vertical velocity (RVV), left horizontal velocity (LHV), and right horizontal velocity (RHV). A moving Pearson correlation coefficient was calculated over a window of 30 consecutive frames to assess the relationship between LVV and RVV, as well as LHV and RHV. The resulting coefficient, termed the vergence reactivity index (VRx), quantified the conjugacy of eye movements within a time window of ∼1 to 2 s. This generated a matrix of VRx values, which was subsequently analyzed.

### 2.3 Transcranial Photobiomodulation Protocol

**Fig. 3:**
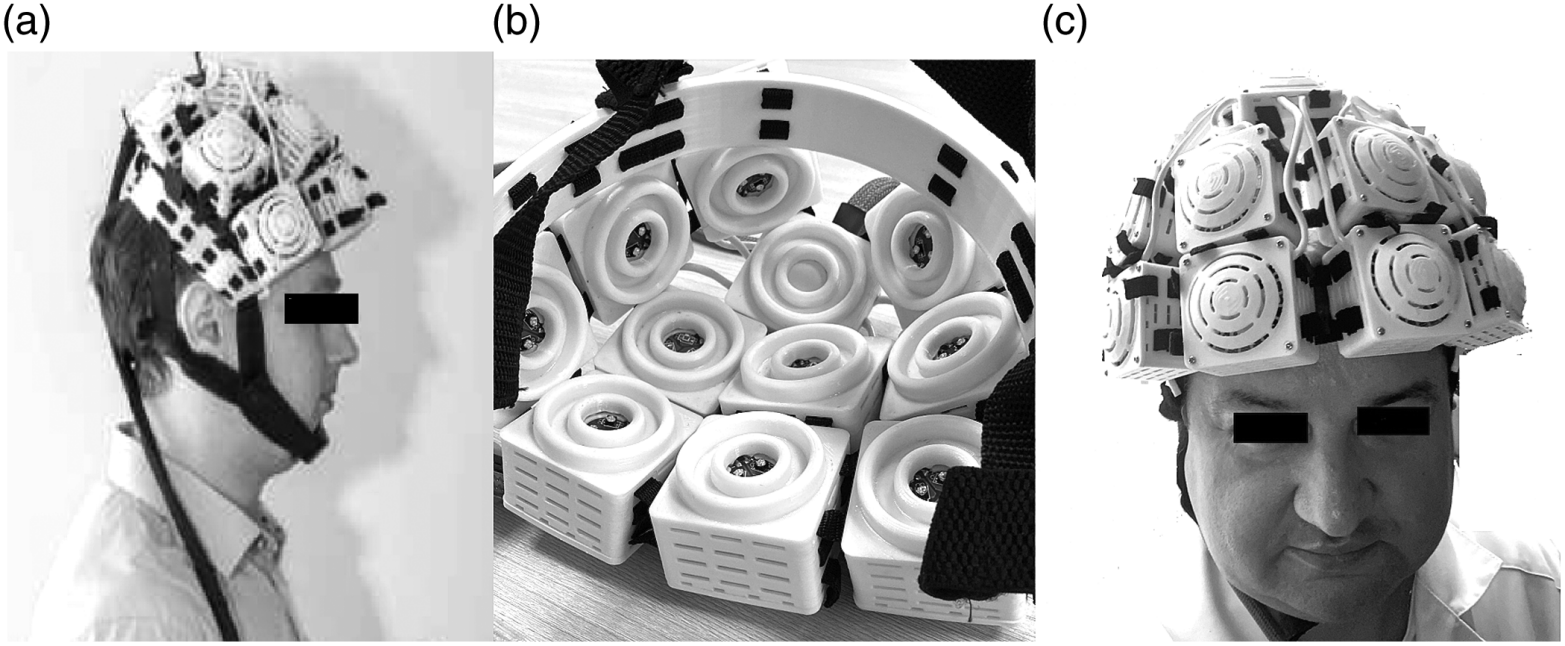
Overview of NIR-TPBM procedure. (a) Lateral view of the NIR helmet (Elmedlife H) placements on the head. (b) Inner view of the NIR-TPBM helmet (10 active NIR laser optodes). (c) Oblique view of the NIR-TPBM appearance.

Transcranial PBM was performed in first group using a low-fluence NIR-TPBM device (Elmedlife H^®^, Nizhny Novgorod, Russia) in the left and right fronto-temporal areas. The NIR-TPBM parameters were as follows: 810-nm laser wavelength; number of NIR optodes, 10; summary output power, 6 W; irradiance, $300\;\mathrm{mW}/{\mathrm{cm}}^{2}$; fluence rate, $103.2\;J/{\mathrm{cm}}^{2}$; light spot area of single optode, $2\;{\mathrm{cm}}^{2}$; the total size of the light delivery, $20\;{\mathrm{cm}}^{2}$ in both fronto-temporal areas; and duration, 10 min. Bilateral geometry and an overview of the NIR-TPBM helmet are shown in Fig. 3.

In the second group, participants wore the NIR-TPBM helmet, but the NIR-TPBM mode remained deactivated. The subject did not know whether the helmet was on because the infrared spectrum is invisible to humans. The duration of the NIR-TPBM and sham was the same.

### 2.4 Statistical Analysis

The software package Statistica 12.0 (TIBCO Inc., United States) was used for data analysis. Data were not normally distributed and expressed as a median [interquartile range]. The VRx were calculated in a linear moving Pearson’s correlation among 30 consecutive values in a 1.5-s time window, averaging the corresponding left and right angular velocities. Statistical analysis was performed using the T-criterion Wilcoxon. The significance level was preset at p<0.05.

## 3 Results

**Fig. 4:**
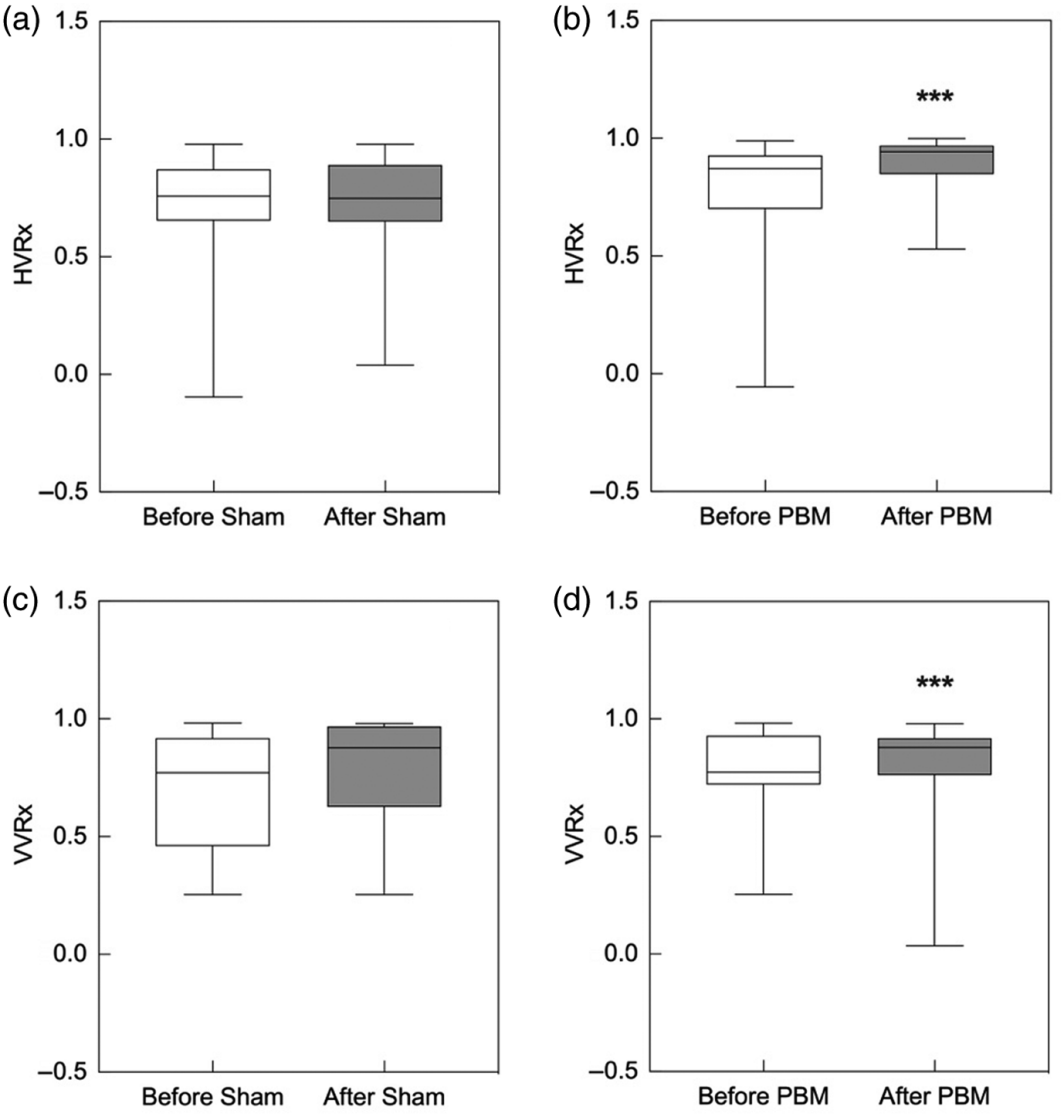
(a) Comparison of vertical VRx values before and after transcranial photobiomodulation. (b) Comparison of horizontal VRx values before and after transcranial photobiomodulation. (c) Comparison of vertical VRx values before sham procedures. (d) Comparison of horizontal VRx values after sham procedures in young, healthy volunteers.

The obtained results are summarized in Table 1. In the first group, NIR-TPBM procedures led to a significant increase in both (vertical and horizontal) VRx. Vertical VRx values after NIR-TPBM were significantly higher than before the procedure: 0.879 [0.761, 0.918] versus 0.774 [0.721, 0.929], p<0.001 (Fig. 4). Similarly, horizontal VRx values after NIR-TPBM were significantly greater than pre-procedure values: 0.943 [0.848, 0.969] versus 0.772 [0.651, 0.890], p<0.001.

**Table 1:** Comparison of the obtained values of the studied parameters.

	Group 1	Group 2	Δ	P
I. VVRx before procedure	0.774 [0.721, 0.929]	0.772 [0.461, 0.919]	−0.002	>0.05
II. HVRx before procedure	0.772 [0.651, 0.890]	0.759 [0.656, 0.871]	0.013	>0.05
III. VVRx after procedure	0.879 [0.761, 0.918]	0.791[0.626, 0.928]	0.088	<0.0001^*^
IV. HVRx after procedure	0.943 [0.848, 0.969]	0.748 [0.651, 0.890]	0.195	<0.0001^*^
V. LVV before procedure	0.131 [−0.584, 1.043]	0.072 [0.009, 0.147]	0.059	>0.05
VI. RVV before procedure	0.052 [−1.174, 1.563]	0.007 [−0.077, 0.057]	0.045	>0.05
VII. LHV before procedure	0.261 [−0.782, 1.046]	0.082 [0.011, 0.163]	0.179	<0.05^*^
VIII. RHV before procedure	0.391 [−0.933, 1.396]	0.029 [−0.034, 0.105]	0.362	<0.05^*^
IX LVV after procedure	0.210 [0.008, 0.368]	0.071 [0.010, 0.142]	0.139	<0.05^*^
X. RVV after procedure	0.012 [−0.076, 0.114]	0.002 [−0.067, 0.054]	0.010	>0.05
XI. LHV after procedure	0.175 [0.002, 0.351]	0.072 [0.001, 0.158]	0.103	>0.05
XII. RHV after procedure	0.034 [−0.003, 0.105]	0.035 [−0.022, 0.091]	− 0.001	>0.05
XIII. ΔVVRx	0.105 [0.010, 0.039]	0.018 [0.08, 0.205]	—	<0.05^*^
XIV. ΔHVRx	0.170 [0.095, 0.196]	0.010 [0.007, 0.018]	—	<0.05^*^
*P* (I to III)	<0.0001^*^	>0.05		—
*P* (II to IV)	<0.0001^*^	>0.05		—
*P* (V to IX)	>0.05	>0.05		—
*P* (VI to X)	>0.05	>0.05		—
*P* (VII to XI)	>0.05	>0.05		—
*P* (VIII to XII)	<0.0001^*^	>0.05		—

LVV, left vertical velocity (rad/s); RVV, right vertical velocity (rad/s); LHV, left horizontal velocity (rad/s); RHV, right horizontal velocity (rad/s); VVRx, vertical vergence reactivity index; HVRx, horizontal vergence reactivity index; Δ, parameter in group 1 minus parameter in group 2; ΔVVRx, parameter VVRx after procedure minus parameter VVRx before procedure; ΔHVRx, parameter HVRx after procedure minus parameter HVRx before procedure; data are presented as median [interquartile range]. *p<0.05

We found that the within-subject differences in VRx values, specifically the changes in horizontal VRx (ΔHVRx) and vertical VRx (ΔVVRx), measured over the same time intervals before and after the TPBM procedure, were significantly greater in group 1 compared with group 2 (p<0.05).

It should be noted that although the RHV changes in group 1 before and after the procedure were statistically significant, we still cannot explain their nature. It is evident that the gaze became more focused, as shown by the slight tendency to decrease the interquartile range of angular velocities of both eyes after NIR-TPBM. We observed no adverse effects during or after the NIR-TPBM procedure. In the second group, the sham procedure did not lead to any significant changes in angular velocities (LVV, RVV, LHV, and RHV) or any VRx. In addition, no adverse effects from the sham procedures were identified.

## 4 Discussion

The aim of our randomized study was to investigate the changes in oculomotor activity and coupling in healthy volunteers based on the assessment of eye movement coupling in response to NIR-TPBM.

To assess the changes in this function, we used the dynamics of the vergence reactivity index (VRx), which has shown its effectiveness in assessing changes in ocular vergence in patients with traumatic brain injury^21^ and SARS-CoV-2.^22^

This study showed that TPBM improves the coupling of oculomotor activity, whereas no changes were detected in the control group.

**Fig. 5:**
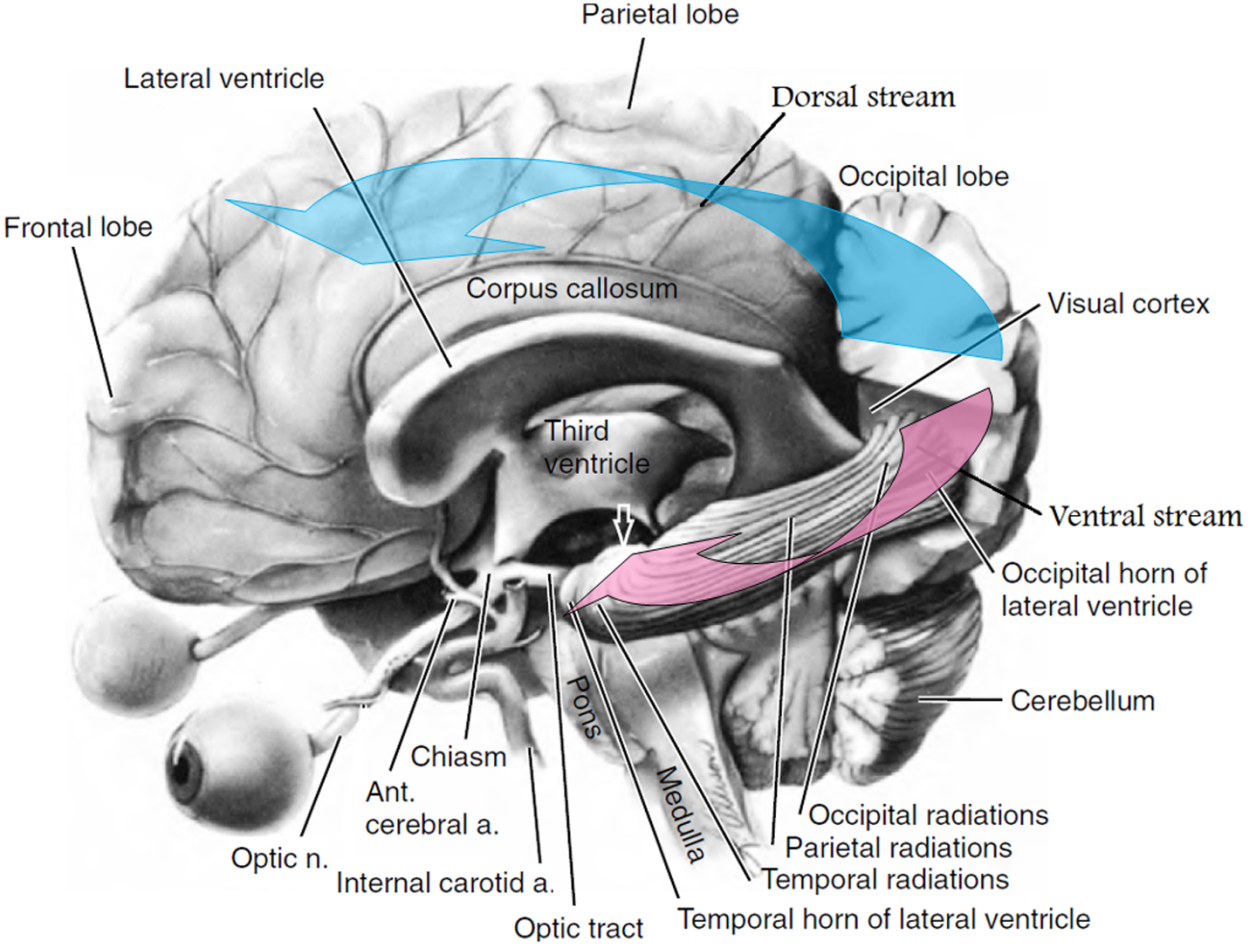
Schematic anatomo-functional organization of the visual pathways (the brain’s left hemisphere was particularly removed). The blue arrow indicates the dorsal stream, and the red arrow indicates the ventral stream. The white arrow indicates the terminal part of the ventral stream. The blue arrow points to regions of the dorsal stream (“WHERE”) involved in visual motion and visuomotor processing.^33^ The effective connectivity of the human dorsal visual cortex stream (“WHERE”) reaches both parietal lobes and then the parahippocampal region. In addition, the dorsal stream structures perform transformations from the retinal coordinate grid to spatial coordinates via conjugate eye movements. The red arrow shows how the ventral visual stream provides “WHAT” input via the external parahippocampal gyrus and how the visual cortex is connected to the orbitofrontal cortex via the temporal regions of the visual analyzer (temporal radiation). The ventral optic stream also has connections with areas in the superior temporal gyrus and inferior parietal visual areas. Modified from Thurtell and Rucker.^34^

Although numerous studies of structural connections in the brain using functional MRI, magnetoencephalography, and electroencephalography have shown that eye movement coupling does not have a strictly determined localization in the brain,^23^^–^^25^ the rate of development of changes in oculomotor activity and coupling after TBI suggests that the modulation affected either the structures directly involved in such coupling or functionally related to them. It is worth noting that although the eye muscles were outside the zone of NIR influence, the oculomotor center in the motor cortex of the frontal lobe was within its reach. Thus, one of the reasons for the identified changes is the direct modulation of the function of the frontal representation of eye position control, which is consistent with previous studies.^26^ On the other hand, according to functional magnetic resonance imaging data, NIR-TPBM causes an increase in the number of functional connections among different parts of the brain (the so-called functional connectivity), including among the abovementioned neural networks of the brain.^27^^,^^28^ Several studies have shown that these networks are of systemic importance in oculomotor control processes^29^^–^^31^ and are anatomically connected by bundles of cortico-collicular axons.^32^ This allowed us to combine them into a dorsal frontoparietal network (the so-called dorsal stream), which has bilateral localization and is activated in a process that requires visual attention and determining the direction of the tracking object (the “WHERE” process) (Fig. 5).^25^

The blue arrow points to regions of the dorsal stream (“WHERE”) involved in visual motion and visuomotor processing.^33^ The effective connectivity of the human dorsal visual cortex stream (“WHERE”) reaches both parietal lobes and then the parahippocampal region. In addition, dorsal stream structures perform transformations from the retinal coordinate grid to spatial coordinates via conjugate eye movements.

Thus, NIR modulation of the networks included in the dorsal stream (DMN, SN, and central executive network) can also be the cause of the improvement in the connectivity of oculomotor activity.

This is confirmed by the fact that an increase in VRx was noted both during smooth pursuits and during saccades, in the formation of which it participates, which is consistent with previous studies.^35^

It should be noted that the dorsal frontoparietal network and its tracts (dorsal stream) are comparable in volume in both hemispheres. Thus, the functioning of the dorsal is not associated with the right- or left-handedness of the subject, unlike the networks included in the so-called ventral stream (the “WHAT” stream).^36^ However, despite this, only right-handed volunteers were examined in our study.

Although we did not evaluate the influence of PBM on the ventral stream, eye tracking was supplemented by an auditory phenomenon of a “single click,” similar to the sound of a camera shutter for each snapshot, and at a speed of 20 frames per second, and was perceived as a “chirping.” Such a paradigm (1) complemented the focus of attention of the subjects and (2) could involve the auditory component of the ventral stream in the exploration process, as shown by Rauschecker.^37^

It should be noted that the relative simplicity of the study paradigm (passive tracking of a stimulus on the eye tracker screen) made it possible to neglect to some extent the errors associated with disparity, in particular, by calculating the moving Pearson’s correlation coefficient among the previous 30 measurements of angular velocities of the eyes, rather than the coordinates of the pupils in space. Thus, eye tracking was used to directly assess oculomotor activity, although this occurred, among other things, through the analysis of the perception of visual images.

Our results on the increase in eye movement coupling after NIR-TPBM, with a high probability, indicate a positive effect on the functional connectivity of neural networks, which opens up broad prospects for its use in various neurological and mental disorders.^38^ Finally, although this study demonstrated the effectiveness of NIR-TPBM on visual pathways function, the single application certainly limits the generalizability of these results to clinical settings. Further researches are needed.

## 5 Conclusion

NIR-TPBM enhances visual pathways function by increasing both vergence reactivity indices in young healthy volunteers.

## Data Availability

The data supporting this study’s findings are available from the first author, AT, upon reasonable request.

## References

[1] Whelan H. T., “The NASA light-emitting diode medical program—progress in space flight and terrestrial applications,” in Proc. AIP Conf. Proc., Vol. 504, pp. 37–43, AIP Publishing, College Park (2003). 10.1063/1.1302454

[2] Hamblin M. R. et al., Low-Level Light Therapy: Photobiomodulation, SPIE Press, Bellingham, Washington, USA (2018).

[3] Vargas E. et al., “Beneficial neurocognitive effects of transcranial laser in older adults,” Lasers Med. Sci. 32(5), 1153–1162 (2017). 10.1007/s10103-017-2221-y 28466195 PMC6802936

[r4] da Silva R. C. M. et al., “Adjunctive photobiomodulation to basic periodontal therapy using different low-power laser application techniques: a systematic review and meta-analysis,” Lasers Med. Sci. 39(1), 207 (2024). 10.1007/s10103-024-04148-2 39093490

[r5] Hao J. et al., “Effects of high-intensity laser therapy on subacromial impingement syndrome: a systematic review and meta-analysis,” Lasers Med. Sci. 39(1), 240 (2024). 10.1007/s10103-024-04190-0 39317844

[r6] Youssef M. et al., “Efficacy of repeated low-level red light (RLRL) therapy on myopia outcomes in children: a systematic review and meta-analysis,” BMC Ophthalmol. 24(1), 78 (2024). 10.1186/s12886-024-03337-5 38378527 PMC10877869

[7] Borges N. C. S. et al., “Photobiomodulation using red and infrared spectrum light emitting-diode (LED) for the healing of diabetic foot ulcers: a controlled randomized clinical trial,” Lasers Med. Sci. 39(1), 253 (2024). 10.1007/s10103-024-04199-5 39382587

[8] Dos Santos S.A. et al., “Effects of photobiomodulation therapy on oxidative stress in muscle injury animal models: a systematic review,” Oxid. Med. Cell. Longev. 2017, 5273403 (2017). 10.1155/2017/5273403 29075364 PMC5623775

[9] Dompe C. et al., “Photobiomodulation-underlying mechanism and clinical applications,” J. Clin. Med. 9(6), 1724 (2020). 10.3390/jcm9061724 32503238 PMC7356229

[10] Salehpour F. et al., “Transcranial photobiomodulation improves cognitive performance in young healthy adults: a systematic review and meta-analysis,” Photobiomodul. Photomed. Laser Surg. 37(10), 635–643 (2019). 10.1089/photob.2019.4673 31549906 PMC6818490

[11] Hennessy M. Hamblin M. R., “Photobiomodulation and the brain: a new paradigm,” J. Opt. 19(1), 013003 (2017). 10.1088/2040-8986/19/1/013003 28580093 PMC5448311

[12] Gilmore J. Knickmeyer R. Gao W., “Imaging structural and functional brain development in early childhood,” Nat. Rev. Neurosci. 19, 123–137 (2018). NRNAAN 1471-003X 10.1038/nrn.2018.1 29449712 PMC5987539

[13] Laude A. et al., “Eye gaze tracking and its relationship with visual acuity, central visual field and age-related macular degeneration features,” Invest. Ophthalmol. Vis. Sci. 59(9), 1264 (2018). IOVSDA 0146-0404

[14] Schimmelpfennig J. et al., “The role of the salience network in cognitive and affective deficits,” Front. Hum. Neurosci. 17, 1133367 (2023). 10.3389/fnhum.2023.1133367 37020493 PMC10067884

[15] Wang X. et al., “Proceedings # 18. Transcranial infrared brain stimulation modulates EEG alpha power,” Brain Stimul.: Basic Transl. Clin. Res. Neuromodul. 10(4), e67–e69 (2017). 10.1016/j.brs.2017.04.111

[16] Barrett D. W. Gonzalez-Lima F., “Transcranial infrared laser stimulation produces beneficial cognitive and emotional effects in humans,” Neuroscience 230, 13–23 (2013). 10.1016/j.neuroscience.2012.11.016 23200785

[17] Xuan W. et al., “Transcranial low-level laser therapy enhances learning, memory, and neuroprogenitor cells after traumatic brain injury in mice,” J. Biomed. Opt. 19, 108003 (2014). JBOPFO 1083-3668 10.1117/1.JBO.19.10.108003 25292167 PMC4189010

[18] Shirkavand A. Akhavan Tavakoli M. Ebrahimpour Z., “A brief review of low-level light therapy in depression disorder,” J. Lasers Med. Sci. 14, e55 (2023). 10.34172/jlms.2023.55 38028864 PMC10658118

[19] da Silva Soares R. Jr. et al., “Applying functional near-infrared spectroscopy and eye-tracking in a naturalistic educational environment to investigate physiological aspects that underlie the cognitive effort of children during mental rotation tests,” Front. Hum. Neurosci. 16, 889806 (2022). 10.3389/fnhum.2022.889806 36072886 PMC9442578

[20] https://www.randomizer.org.

[21] Trofimov A. et al., “Eye tracking parameters correlate with the level of cerebral oxygen saturation in mild traumatic brain injury: a preliminary study,” Adv. Exp. Med. Biol. 1395, 151–156 (2022). AEMBAP 0065-2598 10.1007/978-3-031-14190-4_26 36527630 PMC10042481

[22] Trofimov A. et al., “Comparison of eye-tracking parameters and brain oxygen saturation in patients with COVID-19 moderate pneumonia,” Adv. Exp. Med. Biol. 1463, 141–145 (2024). AEMBAP 0065-2598 10.1007/978-3-031-67458-7_24 39400814 PMC12447872

[23] Corbetta M. Shulman G. L., “Control of goal-directed and stimulus-driven attention in the brain,” Nat. Rev. Neurosci. 3(3), 201–215 (2002). NRNAAN 1471-003X 10.1038/nrn755 11994752

[r24] Parr T. et al., “Dynamic causal modeling of active vision,” J. Neurosci. 39(32), 6265–6275 (2019). JNRSDS 0270-6474 10.1523/JNEUROSCI.2459-18.2019 31182633 PMC6687902

[25] Vossel S. et al., “Deconstructing the architecture of dorsal and ventral attention systems with dynamic causal modeling,” J. Neurosci. 32, 10637–10648 (2012). JNRSDS 0270-6474 10.1523/JNEUROSCI.0414-12.2012 22855813 PMC3432566

[26] Sajad A. et al., “Visual–motor transformations within frontal eye fields during head-unrestrained gaze shifts in the monkey,” Cereb. Cortex 25, 3932–3952 (2015). 53OPAV 1047-3211 10.1093/cercor/bhu279 25491118 PMC4585524

[27] Naeser M. A. et al., “Increased functional connectivity within intrinsic neural networks in chronic stroke following treatment with red/near-infrared transcranial photobiomodulation: case series with improved naming in aphasia,” Photobiomodul. Photomed. Laser Surg. 38, 115–131 (2020). 10.1089/photob.2019.4630 31621498

[28] Naeser M. A. et al., “Transcranial photobiomodulation treatment: significant improvements in four ex-football players with possible chronic traumatic encephalopathy,” J. Alzheimers Dis. Rep. 7(1), 77–105 (2023). 10.3233/ADR-220022 36777329 PMC9912826

[29] Zerbi V. et al., “Rapid reconfiguration of the functional connectome after chemogenetic locus coeruleus activation,” Neuron 103, 702–718.e5 (2019). NERNET 0896-6273 10.1016/j.neuron.2019.05.034 31227310

[r30] Xie X. et al., “Emergence of canonical functional networks from the structural connectome,” NeuroImage 237, 118190 (2021). NEIMEF 1053-8119 10.1016/j.neuroimage.2021.118190 34022382 PMC8451304

[31] Li Y. et al., “Functional connectivity of the central autonomic and default mode networks represent neural correlates and predictors of individual personality,” J. Neurosci. Res. 100, 2187–2200 (2022). JNREDK 0360-4012 10.1002/jnr.25121 36069656

[32] Gaymard B. Lynch J. Ploner C. J., “The parieto-collicular pathway: anatomical location and contribution to saccade generation,” Eur. J. Neurosci. 17(7), 1518–1526 (2003). EJONEI 0953-816X 10.1046/j.1460-9568.2003.02570.x 12713655

[33] Rolls E. T., “Two what, two where, visual cortical streams in humans,” Neurosci. Biobehav. Rev. 160, 105650 (2024). NBREDE 0149-7634 10.1016/j.neubiorev.2024.105650 38574782

[34] Thurtell M. J. Rucker J. C., “Neuro-ophthalmology,” in Youmans and Winn Neurological Surgery, 8th ed., Winn H. R., Ed., p. 200.e8, Elsevier, Philadelphia, PA (2023).

[35] Mishkin M. et al., “Object vision and spatial vision: two cortical pathways,” Trends Neurosci. 6, 414–417 (1983). TNSCDR 0166-2236 10.1016/0166-2236(83)90190-X

[36] Goodale M. Milner A., “Two visual pathways—where have they taken us and where will they lead in the future?” Cortex 98, 283–292 (2018). 10.1016/j.cortex.2017.12.002 29288012

[37] Rauschecker J., “Ventral and dorsal streams in the evolution of speech and language,” Front. Evol. Neurosci. 4, 7 (2012). 10.3389/fnevo.2012.00007 22615693 PMC3351753

[38] Zeng J. et al., “Can transcranial photobiomodulation improve cognitive function in TBI patients? A systematic review,” Front. Psychol. 15, 1378570 (2024).1664-1078 10.3389/fpsyg.2024.1378570 38952831 PMC11215173

